# Bronchopulmonary Dysplasia Definition Based on Respiratory Severity Score

**DOI:** 10.1002/ppul.71750

**Published:** 2026-07-20

**Authors:** Dinushan C. Kaluarachchi, Patrick J. Peebles, Michael R. Lasarev, Scott O. Guthrie, Matthew M. Laughon

**Affiliations:** ^1^ Department of Pediatrics University of Wisconsin – Madison Madison Wisconsin USA; ^2^ Department of Biostatistics and Medical Informatics University of Wisconsin – Madison Madison Wisconsin USA; ^3^ Department of Pediatrics University of Tennessee College of Medicine Chattanooga Tennessee USA; ^4^ Department of Pediatrics University of North Carolina Chapell Hill North Carolina USA

## Conflicts of Interest

Dr. Kaluarachchi and Dr. Guthrie serve as consultants for ONY Biotech Inc. The remaining authors declare that the research was conducted in the absence of any commercial or financial relationships that could be construed as a potential conflict of interest.

To the Editor,

1

Despite being a common complication of extreme prematurity, defining and grading bronchopulmonary dysplasia (BPD) has been a challenge. Over the last several decades, various definitions have been developed, yet none have proven to provide sufficient prognostic accuracy [1]. BPD definition by Jensen et al is one of the latest attempts to define BPD [2]. This grading system is based on the type of respiratory support delivered to the infant at 36 weeks postmenstrual age (PMA). A definition that focuses only on respiratory support type may not reflect true respiratory illness severity, as it does not include the degree of support, such as mean airway pressure (MAP) or the fraction of inspired oxygen (FiO2). In this study, we evaluate a novel BPD grading system based on the respiratory severity score (RSS), which accounts for both MAP and FiO2. The objective of the current study is to evaluate the performance of a BPD definition based on RSS (MAP x FiO2) at 36 weeks PMA in predicting severe respiratory morbidity severity (RMS) compared to the BPD definition by Jensen et al. [2].

## 2 Methods

This is a retrospective cohort study of preterm infants born at 23 0/7 to 28 6/7 weeks' gestation from the Prematurity and Respiratory Outcomes Program (PROP, *n* = 835) [3]. Deaths before 36 weeks PMA (*n* = 63), withdrawals (*n* = 7), infants who were transferred prior to 36 weeks (*n* = 16), inadequate data to calculate RSS (*n* = 10), and unknown RMS status (*n* = 56) were excluded. RMS was defined according to the original study criteria(Table 1) [3]. BPD grading was defined according to two BPD definitions: (1) the definition by Jensen et al based on the type of respiratory support at 36 0/7 weeks; (2) the proposed BPD definition based on average RSS at 36 weeks PMA (Table 1). Each patient's daily RSS was documented at 12 noon on each day for the week of 36 weeks' PMA (36 0/7 to 36 6/7) and the average RSS for 36 weeks' PMA was calculated. Daily RSS was calculated or estimated as follows: continuous positive airway pressure, non‐invasive positive pressure ventilation, invasive ventilation (RSS = MAP x FiO2); high‐flow nasal cannula or nasal cannula with flow 1 L/min–10 L/min (RSS = flow in L/min x FiO2); nasal cannula flow < 1 L/min (RSS = 0.25). We proposed three RSS‐based BPD grades where nonzero average RSS values at 36 weeks' PMA were divided into three tertiles to create BPD grades. Log‐binomial regression was used to determine whether the risk of severe RMS was associated with the severity of BPD according to Jensen's definition or with the RSS‐based BPD definition. The Concordance index (C‐index) was calculated to assist in evaluating how successful each definition was in predicting severe RMS. DeLong's test was used to compare C‐indexes between models. Informed consent was obtained for participation in the original PROP cohort. The University of Wisconsin – Madison institutional review board determined that this study is not considered research involving human subjects as defined by the Department of Health and Human Services and Food and Drug Administration regulations.

**Table 1:** Bronchopulmonary dysplasia (BPD) and respiratory morbidity severity (RMS) definitions.

BPD or RMS	Grade/Severity	Criteria
BPD at 36 0/7 weeks postmenstrual age according to the BPD definition by Jensen et al.	Jensen No BPD	No respiratory support
	Jensen Grade 1 BPD	Nasal cannula < = 2 L/min
	Jensen Grade 2 BPD	Nasal cannula > 2 L/min or noninvasive positive airway pressure
	Jensen Grade 3 BPD	Invasive mechanical ventilation
BPD definition based on the average Respiratory Severity Score (RSS) at 36 weeks postmenstrual age	RSS‐based No BPD	RSS = 0
	RSS‐based Grade 1 BPD	RSS first tertile (RSS 0.01–0.25)
	RSS‐based Grade 2 BPD	RSS second tertile (RSS 0.26‐0.64)
	RSS‐based Grade 3 BPD	RSS third tertile (RSS > = 0.65)
RMS	Severe	If there were ≥ 2 respiratory hospitalizations, home supplemental oxygen after 3 months or any home mechanical ventilation, systemic steroid exposure or pulmonary vasodilators at any time, or symptoms despite concurrent inhaled corticosteroids in ≥ 2 questionnaires. Infants who died secondary to a cardiopulmonary cause were classified as severe RMS
	Moderate/Mild	if 1 hospitalization, home oxygen < 3 months' corrected age, or tracheostomy without ventilation, any inhaled corticosteroid exposure, or bronchodilator or symptoms in ≥ 2 questionnaires
	Minimal/None	all other cases.

## 3 Results

The study included 683 infants. Average RSS over 36 weeks' PMA ranged from zero (294/683; 43.0%) to a maximum of 19.7. Non‐zero average RSS values were grouped into three tertiles as follows: RSS BPD grade 1 (RSS 0.01–0.25), RSS BPD grade 2 (RSS 0.26–0.64), and RSS BPD grade 3 (RSS > = 0.65). RSS BPD grade 1 included a higher proportion of infants compared to RSS BPD grades 2 and 3 due to having a great many values that are “tied”, particularly at the value of RSS = 0.25 that also happens to be the first tertile. There was a strong correlation between the two BPD grading systems (Kendall's tau = 0.87, 95% CI: 0.84–0.89). Log‐binomial regression revealed that the risk of severe RMS was considerably elevated in each non‐zero level of BPD for both definitions (*p* < 0.001 for each grade). C ‐indices treating each definition as an ordered factor, as well as assuming a linear trend with respect to the BPD definition by Jensen et al, all had the same ability to predict infants with severe RMS (C = 0.67; 95% CI: 0.63–0.71 for each) with no difference noted between the two definitions (*p* = 0.89) (Table 2).

**Table 2:** Estimated risk ratios (RR) and 95% confidence intervals (CI) for severe respiratory morbidity severity according to the Bronchopulmonary Dysplasia (BPD) definition by Jensen et al. and the BPD definition based on respiratory severity score (RSS). Concordance measures are reported for each model.

	Risk	Risk ratio (95% CI)	C‐index (95% CI)
BPD definition by Jensen et al.			
Jensen No BPD	67/309 (0.22)	Ref	0.67 (0.63–0.71)
Jensen Grade 1 BPD	94/263 (0.36)	1.65 (1.26, 2.15)	
Jensen Grade 2 BPD	49/82 (0.60)	2.76 (2.08, 3.61)	
Jensen Grade 3 BPD	25/29 (0.86)	3.98 (2.99, 5.09)	
RSS‐based BPD definition			
RSS‐based No BPD	59/294 (0.20)	Ref	0.67 (0.63–0.71)
RSS‐based Grade 1 BPD	65/166 (0.39)	1.95 (1.45, 2.62)	
RSS‐based Grade 2 BPD	35/95 (0.36)	1.84 (1.29, 2.58)	
RSS‐based Grade 3 BPD	76/128 (0.59)	2.96 (2.26, 3.87)	

## 4 Discussion

Using data from a large multi‐center cohort of preterm infants, we demonstrated that an RSS‐based BPD grading system had the same ability to predict severe RMS compared to the BPD grading system by Jensen et al. Given the simplicity of the BPD grading system by Jensen et al., we see no added advantage of the calculated RSS definition since it demonstrates comparable prognostic accuracy.

However, there remains a need for a definition of BPD that is robust to evolving respiratory management strategies over time. The use of respiratory support and oxygen supplementation in convalescent preterm infants may reflect clinician practice patterns and non‐respiratory clinical factors, including apnea of prematurity or upper airway abnormalities. Consequently, continuing to explore more objective measures of lung function may provide a more reliable basis for defining BPD. Finally, the timing of BPD assessment warrants reconsideration, as emerging evidence suggests that determination at 40 weeks PMA may be more appropriate for contemporary preterm populations than the traditional 36‐week benchmark [4].

## 5 Conclusion

There was a strong correlation between the RSS‐based BPD grading system and the BPD grading according to Jensen et al., leading to no difference in performance of these two measures for predicting severe RMS. Further research is needed to define BPD with criteria that are resistant to changes in clinical practice over time and better suit modern‐day extreme preterm infants.

## Author Contributions

**Dinushan C. Kaluarachchi:** conceptualization, investigation, writing – original draft, methodology. **Patrick J. Peebles:** methodology, conceptualization, investigation, writing – review and editing. **Michael R. Lasarev:** writing – review and editing, methodology, formal analysis, investigation, data curation. **Scott O. Guthrie:** investigation, methodology, writing – review and editing. **Matthew M. Laughon:** investigation, methodology, writing – review and editing; supervision.

## Funding

The authors have nothing to report.

## Data Availability

The data that support the findings of this study are available from the corresponding author upon reasonable request.
